# Secretory Leukoprotease Inhibitor (Slpi) Expression Is Required for Educating Murine Dendritic Cells Inflammatory Response Following Quercetin Exposure

**DOI:** 10.3390/nu9070706

**Published:** 2017-07-06

**Authors:** Stefania De Santis, Vanessa Galleggiante, Letizia Scandiffio, Marina Liso, Eduardo Sommella, Anastasia Sobolewski, Vito Spilotro, Aldo Pinto, Pietro Campiglia, Grazia Serino, Angelo Santino, Maria Notarnicola, Marcello Chieppa

**Affiliations:** 1 National Institute of Gastroenterology “S. de Bellis”, Institute of Research, Via Turi, 27, 70013 Castellana Grotte, Italy; stefania.desantis@ispa.cnr.it (S.D.S.); vanessa.galleggiante@libero.it (V.G.); lety.scandiffio@hotmail.it (L.S.); marinaliso@libero.it (M.L.); vito.spilotro@irccsdebellis.it (V.S.); grazia.serino@irccsdebellis.it (G.S.); 2 Institute of Sciences of Food Production C.N.R., Unit of Lecce, via Monteroni, 73100 Lecce, Italy; angelo.santino@ispa.cnr.it; 3 Department of Pharmacy, School of Pharmacy, University of Salerno, 84084 Fisciano, Italy; esommella@unisa.it (E.S.); pintoal@unisa.it (A.P.); pcampiglia@unisa.it (P.C.); 4 University of East Anglia, Norwich Research Park, Norwich NR4 7TJ, UK; a.sobolewski@uea.ac.uk; 5 European Biomedical Research Institute of Salerno (EBRIS), Via S. de Renzi, 3, 84125 Salerno, Italy

**Keywords:** quercetin, dendritic cells, Slpi, inflammation

## Abstract

Dendritic cells’ (DCs) ability to present antigens and initiate the adaptive immune response confers them a pivotal role in immunological defense against hostile infection and, at the same time, immunological tolerance towards harmless components of the microbiota. Food products can modulate the inflammatory status of intestinal DCs. Among nutritionally-derived products, we investigated the ability of quercetin to suppress inflammatory cytokines secretion, antigen presentation, and DCs migration towards the draining lymph nodes. We recently identified the Slpi expression as a crucial checkpoint required for the quercetin-induced inflammatory suppression. Here we demonstrate that Slpi-KO DCs secrete a unique panel of cytokines and chemokines following quercetin exposure. In vivo, quercetin-enriched food is able to induce Slpi expression in the ileum, while little effects are detectable in the duodenum. Furthermore, Slpi expressing cells are more frequent at the tip compared to the base of the intestinal villi, suggesting that quercetin exposure could be more efficient for DCs projecting periscopes in the intestinal lumen. These data suggest that quercetin-enriched nutritional regimes may be efficient for suppressing inflammatory syndromes affecting the ileo-colonic tract.

## 1. Introduction

Quercetin is among the best known phytochemicals able to impact different aspects of human health [1]. Quercetin also has anti-oxidant [2,3] anti-proliferative [4,5,6] and anti-inflammatory properties [7,8,9]. We recently demonstrated that murine dendritic cells, previously exposed to quercetin, suppress inflammatory pathways induced by LPS administration [10,11,12].

Dendritic cells (DCs) are the most powerful antigen presenting cells and are able to capture antigens, migrate to the draining lymph node and present them to initiate the adaptive immune response [13,14]. DCs were long considered to be pro-inflammatory in the mature state and anti-inflammatory in the immature state [15,16]. Only recently have tolerogenic DCs been characterized [17,18,19]. In peripheral tissues, the inflammatory abilities of DC progenitors are dynamically regulated by the local milieu. Several factors may help imprint DCs tolerance, particularly in an anatomical compartment exposed to a large variety of antigens [20,21,22]. In the intestine, incoming progenitors adapt to the temporary need of the tissue, usually becoming inflammatory-impaired DCs; nonetheless, traumatic events and/or hostile infections can change the intestinal milieu that may become inflammatory permissive [23]. DCs polarization in the intestine represents a paradigm for the induction of tolerogenic DCs—a dynamic imprinting mediated by the host secreted factors [24], the microbiome [25,26], and nutritionally-derived factors [27].

In the small intestine, DCs can sample luminal antigen by projecting “periscopes” through the epithelial monolayer into the intestinal lumen [28,29,30,31]. Due to the mucus and antimicrobial protein gradient, the tip of the villi are more frequently exposed to luminal antigens, and consequently, DCs periscopes are more frequent in this region [29].

We already demonstrated that Slpi is required for quercetin-mediated immune suppression [32]. Quercetin exposure can induce Slpi expression even in the absence of inflammatory insult, but little is known about the inflammatory pathway regulated by Slpi. Here we further explored the axis between quercetin-exposed DCs, inflammasome secretion, and Slpi expression. We then addressed if a quercetin-enriched diet was able to induce Slpi expression along the ileum and colon. Knowing that DCs extensions into the intestinal lumen were more frequent at the tip of the villi, we investigated if the presence of quercetin in the lumen could determine a gradient of Slpi expression from the tip to the base of the villi. Our results indicate that DCs may better sense (and respond to) the intestinal content if located at the tip of the intestinal villi, highlighting the importance of nutritionally-derived bioactive compounds in educating the immune response.

## 2. Materials and Methods

### 2.1. Mice

Ethics Statement: investigation has been conducted according to national and international guidelines and has been approved by the authors’ institutional review board (Commission for Animal Wellbeing—OPBA).

Six-to-eight-week-old male mice were purchased from Jackson Laboratories: Wild-type C57BL/6 (Stock No.: 000664; weight: approximately 20 g) and B6; 129-Slpitm1Smw/J (Stock No.: 010926; weight: approximately 20 g). C57BL/6 and B6; 129-Slpitm1Smw/J mice were fed with either a quercetin-enriched food (0.5%) for 4 weeks using aglycone quercetin powder (FARMALABOR SRL Cat n. 1936) or a standard diet (six mice were used in each group). All animal experiments were carried out in accordance with Directive 86/609 EEC enforced by Italian D.L. n. 26/2014, and approved by the Committee on the Ethics of Animal Experiments of Ministero della Salute—Direzione Generale Sanità Animale (Prot. 2012/00000923 A00: Eo_GINRC and 103/2016-PR) and the official RBM veterinarian. Animals were euthanatized if found in severe clinical condition in order to avoid undue suffering.

### 2.2. Generation and Culture of Murine DCs

Bone marrow derived DCs (BMDCs) were obtained from 6–8 week old male C57BL/6 or Slpi-KO mice. Briefly, a single cell suspension of BMDCs was prepared by flushing the tibiae and femurs with 0.5 mM EDTA followed by hypotonic lysis of red blood cells with ACK (Ammonium-Chloride-Potassium) lysing buffer. Cells were plated in a 10 mL dish (1 × 10^6^ cells/mL) and cultured in RPMI 1640 supplemented with 10% heat-inactivated fetal bovine serum (FBS), 100 U/mL penicillin, 100 mg/mL streptomycin, 25 μg/mL rmGM-CSF and 25 μg/mL rmIL-4 at 37 °C in a humidified 5% CO_2_ atmosphere. On day 5, cells were harvested, restimulated with new growth factors, and plated 1 × 10^6^ cells/mL on a 24-well culture plate. Cells were treated with 25 µM of quercetin on day 5 and day 7. On day 8, BMDCs were stimulated with 1 μg/mL of lipopolysaccharide (LPS) for 24 h.

#### Materials

Cell culture media and antibiotics were obtained from Thermo Fisher Scientific, Waltham, MA, USA. Growth factors were obtained from Miltenyi Biotec, Bergisch Gladbach, Germany. Quercetin and LPS (L6143) were obtained from Sigma-Aldrich, St. Louis, MO, USA.

### 2.3. Multiplex

Cell culture supernatants were analyzed using the Bead-based Multiplex for the Luminex^®^ platform (LaboSpace srl, Via Ranzato, 12—20128 Milan, Italy).

### 2.4. Cytofluorimetric Analysis

FoxP3 staining: Spleen, mesenteric lymph nodes (MLNs) and lamina propria (LP) were isolated from 6-to-8-week old mice fed with standard or quercetin-enriched food. Spleen and MLNs were passed through a 40 µm cell strainer (BD Biosciences, Franklin Lakes, NJ, USA) to obtain a single cells suspension that was washed with DPBS 1X (Gibco, NY, USA) + 0.5% bovine serum albumin (BSA, Sigma-Aldrich, St. Louis, MO, USA). LP T-cell analysis was performed starting from single cell suspension of the ileum. Briefly, Peyer’s Patches were removed, intestinal segments (1 cm long) were washed with 2.5 mM EDTA to remove the epithelial cells and digested with collagenase and DNase (Sigma Aldrich, St. Louis, MO, USA) using the GentleMacs suggested protocol. Single cells suspensions from spleen, MLNs, and LP were stained with CD4 APC-Vio 700 (Miltenyi Biotec, Bergisch Gladbach, Germany). Cells were then permeabilized with Foxp3 Fixation/Permeabilization Kit (eBioscience, San Diego, CA, USA) and subsequently washed with PERM Buffer (eBioscience, San Diego, CA, USA). Finally, cells were stained with Foxp3 PE-Cy5 (Miltenyi Biotec, Bergisch Gladbach, Germany). Flow Cytometer acquisition was performed using NAVIOS (Beckman Coulter, Brea, CA, USA).

T cells Intracellular Staining: T cells from spleen, MLN, and LP from 8-week-old mice fed with standard or quercetin-enriched food were cultured with a 500X Cell Stimulation Cocktail (eBiosceince, San Diego, CA, USA) for 12 h, washed with DPBS 1X + 0.5% BSA and stained with CD4 APC-Vio 700 (Miltenyi Biotec, Bergisch Gladbach, Germany). After washing, cells were then permeabilized with BD CytoFix/CytoPerm^®^ Fixation/Permeabilization Kit^®^ (BD Biosciences, Franklin Lakes, NJ, USA), washed with PERM Buffer, and stained with: IL-17A FITC, IFNγ-APC, TNFα PE and IL-4 APC as per manufacturer’s instructions (Miltenyi Biotec, Bergisch Gladbach, Germany). Flow Cytometer acquisition was performed using NAVIOS.

### 2.5. Laser Capture Microdissection (LCM)

The lamina propria cells from the tip or the base of the ileum were micro-dissected using a Leica CTR 6000 microscope (Leica Microsystems, Wetzlar, Germany). Tissues were explanted and immediately embedded in OCT (VWR International, Radnor, PA, USA) and frozen at −80 °C. Serial sections (10 μm) were cut by a cryostat (TiEsseLab, Milan, Italy) and placed on 4 µm PEN Frame slides (Cod. 11600289, Leica Microsystems, Wetzlar, Germany). Sections were stained with hematoxylin and eosin (H & E) standard protocol and immediately micro-dissected to collect the mRNA.

### 2.6. RNA Extraction and Quantitative PCR (qPCR) Analysis

Total RNA was isolated from the ileum and colon. The RNA was extracted using TRIzol^®^ (Thermo Fisher Scientific, Waltham, MA, USA) according to manufacturer’s instructions. Five hundred ng of total RNA was reverse transcribed with the High Capacity cDNA Reverse Transcription kit (Thermo Fisher Scientific, Waltham, MA, USA) by using random primers for cDNA synthesis. Gene expression of Slpi and Gapdh were determined by using TaqMan Gene Expression Assays (Thermo Fisher Scientific, Waltham, MA, USA)—murine probes: Mm00441530_g1 and Mm99999915_g1, respectively. Real-time analysis performed on CFX96 System (Biorad, Hercules, CA, USA) and the expression of all target genes were calculated relative to GAPDH expression using ΔΔCt method.

### 2.7. Quercetin Quantification from Fecal Samples

Stool and intestinal content samples were thawed, accurately weighed and extracted as follows: Samples were solubilized in Methanol and subjected to ultrasonic bath for 15 min. Subsequently samples were centrifuged at 4 °C for 15 min the process was repeated three times. Supernatants were pooled and dried under reduced pressure. All samples were re-solubilized in methanol (1 mL) filtered on 0.45 µm filters and injected. Homogenized tissue samples with METABOPREP-LC kit (Hosmotic, Piano di Sorrento (NA), Italy) according to producer instructions. Liquid chromatography-tandem mass spectrometry (LC-MS/MS) analyses were performed on a Nexera UHPLC coupled to a triple quadrupole LCMS-8050 (Shimadzu, Milan, Italy). The separation was carried out with a 50 × 2.1 mm, 1.7 μm BEH C18 column (Waters, Milford, MA, USA). Mobile phases were: A) 0.1% CH_3_COOH in Water *v/v*, B) ACN plus 0.1% CH_3_COOH *v/v*. Gradient 0–3.00 min, 10–98% B, hold for 30 s, returning at 10%B in 0.1 s. Column equilibration 2 min. Column oven: 45 °C. MS detection was performed in negative ionization (ESI^−^) in multiple reaction monitoring (MRM): quantifier 301-151, qualifier 301-179. Interface temperature 400 °C, Desolvation line 200 °C, Heat Block 400 °C. Drying gas (N_2_) 10 L/min, Heating gas (air) 10 L/min, Nebulizing gas 3 L/min. Probe voltage −3.5 kV, detector voltage 1.80 kV. Quercetin was quantified by external calibration. A stock solution of quercetin 1 mg/1 mL was prepared in methanol and a seven point calibration curve was built (0.025–70 µg/mL) and triplicate analyses of each point were run; this methodology showed excellent linearity (*R*^2^: 0.999). Quercetin quantification refers to stool samples and intestinal content collected from duodenum, ileum, and colon of mice fed for 28 days with quercetin-enriched food as compared to mice fed with standard food. Additionally, the ileum content was assessed for quercetin levels. For this analysis, the tissue was collected in dH_2_O and homogenized with GentleMACS^®^ (Miltenyi Biotec, Bergisch Gladbach, Germany) by using the program for protein digestion specific for M tubes^®^ (Miltenyi Biotec, Bergisch Gladbach, Germany).

### 2.8. Statistical Analysis

Statistical analysis was performed using the Graphpad Prism statistical software release 5.0 for Windows XP. All data were expressed as means ± SEM of data obtained from at least three independent experiments. We evaluated the statistical significance of the grouped analysis with the two-way ANOVA test using the Bonferroni as a post-test. Results were considered statistically significant at *p* < 0.05.

## 3. Results and Discussion

### 3.1. Quercetin Fails to Reduce Inflammatory Cytokine Secretion in Slpi-KO DCs

**Figure 1:**
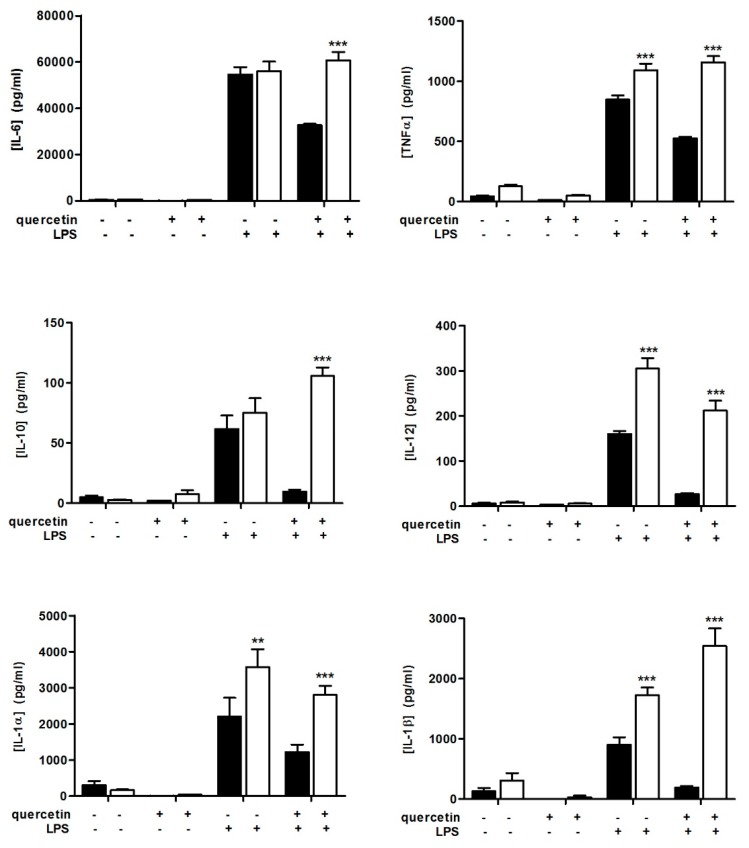
Quercetin reduces inflammatory cytokines secretion in wild-type (WT) dendritic cells (DCs). Bone marrow-derived DCs (BMDCs) were cultured from WT (black bars) and Slpi-KO (white bars) mice, treated with quercetin at day 5 and 7 and exposed to lipopolysaccharide (LPS). Secretion of cytokines was determined 24 h later by Multiplex assay. Bars represent mean concentration of interleukin-6 (IL-6), tumor necrosis factor alpha (TNFα), interleukin-10 (IL-10), interleukin-12 (IL-12), interleukin-1 alpha (IL-1α) and interleukin-1 beta (IL-1β) ± SEM (*n* = 4). *** *p* < 0.001, ** *p* < 0.01.

We recently demonstrated that quercetin-exposed DCs from wild-type (WT) mice reduce their ability to release tumor necrosis factor alpha (TNFα), interleukin 1 alpha, 1 beta, 6, 10 and 12 (IL-1α, IL-1β, IL-6, IL-10, and IL-12) following LPS administration [10,11] and that Slpi expression was a non-redundant checkpoint required for quercetin-mediated TNFα secretion suppression [32]. Here we explored the secretion of IL-1α, IL-1β, IL-6, IL-10, and IL-12 from quercetin-exposed Slpi-KO DCs following LPS administration. Slpi-KO DCs respond to LPS by releasing higher amounts of TNFα, IL-1α, IL-1β, and IL-12. However, quercetin administration failed to reduce inflammatory cytokine secretion in Slpi-KO DCs (Figure 1), which was in contrast to previously observed effects on WT DCs [10,11]. Notably, LPS-induced IL-1β secretion from Slpi-KO DCs was increased following pre-exposure to quercetin (Figure 1). Altogether, these analyses of the secretome confirm the role of quercetin as an anti-inflammatory agent [32], and provide a new insight into how quercetin can exert its effects on IL-1β secretion in the absence of Slpi. The anti-inflammatory effects of quercetin were absent or reduced in the absence of Slpi for all cytokines considered, with the exception of IL-1β, suggesting a different regulatory machinery involved in the secretion of this crucial inflammatory cytokine.

### 3.2. Slpi-KO DCs Fail to Secrete CXCL-1 Independently from Quercetin Exposure

**Figure 2:**
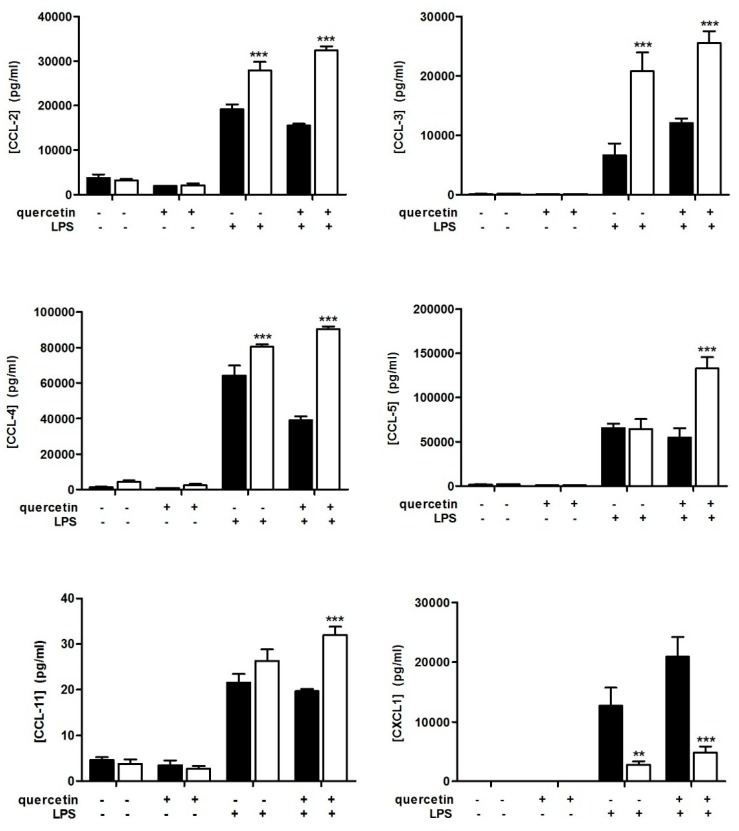
Quercetin exposure and chemokines release. Slpi-KO DCs fail to release the chemokine (C-X-C motif) ligand-1 CXCL-1 following LPS administration independently from quercetin exposure. BMDCs were cultured from WT (black bars) and Slpi-KO (white bars) mice, treated with quercetin at day 5 and 7 and exposed to LPS. Secretion of chemokines was determined after 24 h by Multiplex assay. Bars represent mean concentration of the (C-C motif) ligand-2, -3, -4, -5, -11 (CCL-2, CCL-3, CCL-4, CCL-5, CCL-11) and CXCL-1 ± SEM (*n* = 4). *** *p* < 0.001, ** *p* < 0.01.

The chemokine secretion profile was differentially modulated in WT and Slpi-KO DCs. Slpi-KO cells released higher amounts of CCL-2, CCL-3, and CCL-4 following 24 h LPS stimulation compared to DCs from WT mice. However, quercetin pre-exposure did not affect the secretion of CCL-2, CCL-3, CCL-4, or CCL-11. Of note (and different from other chemokines), CCL-5 secretion was significantly higher in LPS-treated Slpi-KO DCs pre-exposed to quercetin. Strikingly, Slpi-KO DCs failed to release CXCL-1 in response to LPS administration, independent of quercetin exposure (Figure 2). CXCL-1 is a major neutrophil chemoattractant and binds to CXCR2 in mice [33]. This novel observation may be important for future investigation due to the crucial role of CXCL-1 in neutrophil recruitment.

### 3.3. A Quercetin-Enriched Diet Promotes Slpi Expression in the Ileum and Colon

We already demonstrated that quercetin was able to induce Slpi expression in murine colon if administered by gavage [32]. Quercetin is commonly present in fruits and vegetables; thus, we decided to evaluate whether Slpi expression could be induced by providing a quercetin-enriched diet to wild-type mice. Mice received quercetin-enriched or standard diet for 4 weeks. No difference in food consumption or mice weight was registered (data not shown). To evaluate the quantity of quercetin present in the intestinal lumen, we collected the luminal content of the duodenum, ileum, colon, and mice stools following 4 weeks of enriched-diet and assessed quercetin presence by LC-MS analyses. We also collected 0.5 cm of the ileum and quantified quercetin present in the cellular extract. Quercetin concentration was higher in the duodenum and gradually diminished passing from the ileum to the colon (Table 1). These data confirm that quercetin was able to safely pass the stomach and reach the intestinal lumen. Quercetin was detected at a very low concentration in cell extract from the ileum, suggesting local rather than systemic DCs exposure to quercetin in these experimental conditions. Although not direct evidence of quercetin absorption (serum levels of quercetin were below detection in line with what has previously been demonstrated [34]), the following results showing Slpi induction are consistent with at least some of the ingested quercetin being able to induce biological effects in the host.

**Figure 3:**
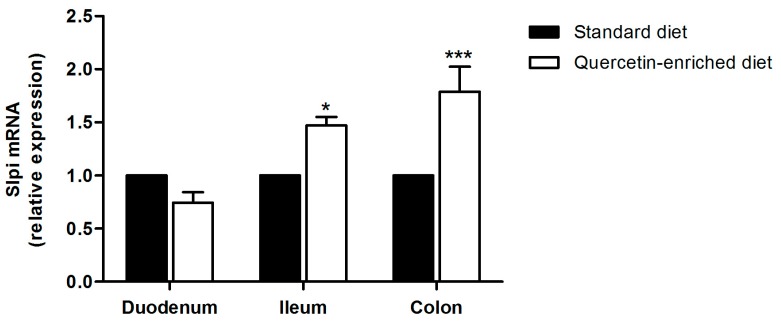
Quercetin induces Slpi expression in the ileum and colon. Quercetin-enriched or standard diet was administered for 4 weeks. Slpi expression was measured by qPCR in the small intestine or colon of standard (black bars) and quercetin (white bars) fed mice. Bars represent mean expression ± SEM (*n* = 3) for each treatment. *** *p* < 0.001, * *p* < 0.05.

**Figure 4:**
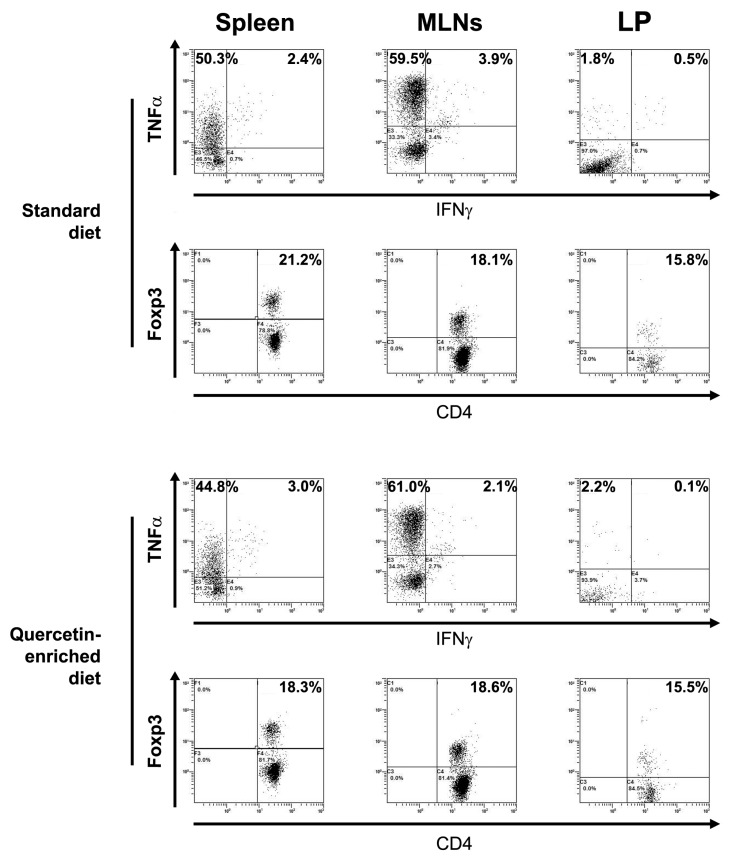
T cell polarization in mesenteric lymph node (MLN), spleen, and lamina propria (LP). Representative intracellular staining of CD4^+^ T cells. No significant differences could be observed in the percentage of TNFα^+^, IFNγ^+^, or Foxp3^+^ cells in mice treated with standard or quercetin-enriched diet (*n* = 3).

Slpi expression was assessed by qPCR in the duodenum, ileum, and colon. A quercetin-enriched diet induced Slpi expression in the ileum and colon, but failed to induce Slpi in the duodenum (Figure 3). These results suggest that Slpi induction was the result of local rather than systemic DCs exposure to quercetin. To investigate if quercetin-enriched food could change the T cell polarization, we performed the intracellular staining of spleen, MLNs, and LP CD4^+^ T cells. Figure 4 shows that no significant difference was detected in the TNFα, IFNγ, and Foxp3 populations, likely due to the absence of an inflammatory insult. Additionally, IL-17A and IL-4 CD4^+^ T cells percentages did not change with the quercetin-enriched diet (data not shown).

Slpi induction observed in the absence of inflammation supports the idea of polyphenol-rich nutritional regimes that may help to prevent chronic inflammatory syndromes.

### 3.4. Quercetin-Mediated Slpi Induction Is Detectable at the Tip of the Villi

**Figure 5:**
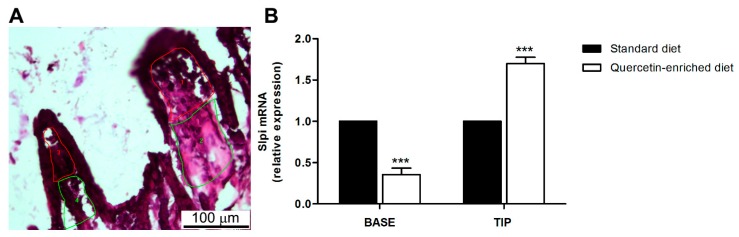
Quercetin induces Slpi expression in the tip of the intestinal villi. Animals were administered quercetin-enriched or standard diet for 4 weeks. (**A**) Representative image demonstrating captured lamina propria cells from the tip (red) or the base (green) of intestinal villi; (**B**) Lamina propria cells from standard (black bars) and quercetin (white bars) diet were laser-captured and Slpi expression measured by qPCR. Bars represent mean expression ± SEM (*n* = 3) for each treatment. *** *p* < 0.001.

In healthy individuals, the mucosal immune system is dynamically educated towards tolerance to luminal antigens, including commensal bacteria and food derived antigens [35]. Several factors contribute to DCs’ “maturation” [23,24], including food-derived products [27]. Intestinal dendritic cells resident in the lamina propria of the villi are able to project periscope-like dendrites into the intestinal lumen and sample luminal content [28,29,30]. As the periscope distribution is mainly concentrated at the tip of the villi, we aimed to detect a correlation between Slpi expression and DCs localization along the villus axis. (Figure 5A). Slpi mRNA expression was increased in samples taken from the tip of the villi in mice fed a quercetin-rich diet (Figure 5B). We acknowledge that this is an indirect indication that sampling DCs encounter quercetin directly in the intestinal lumen and respond to it by up-regulating Slpi. However, it is conceivable that nutritionally-derived factors contribute directly to the intestinal immune cell response by inducing the expression of proteins capable of attenuating the inflammatory response. Indeed, Slpi is also induced by the thymic stromal lymphopoetin (Tslp) [36]—a key factor for intestinal dendritic cell “maturation” [37]. Future studies will further elucidate how nutritional compounds can be used to modulate immune tolerance and mucosal homeostasis through the Slpi–DCs axis, paving the way for the translational use of Slpi-inducing diets for chronic inflammatory syndromes.

## 4. Conclusions

Nutritional regimes enriched in quercetin may create a pro-tolerogenic milieu in the gastrointestinal tract, which could help maintain homeostasis and prevent chronic inflammatory disorders. This work suggests that antigen presenting cells (DCs) residing at the tip of the intestinal villi project dendrites into a quercetin-rich intestinal lumen and subsequently upregulate Slpi expression. Slpi^+^ DCs are able to suppress the inflammatory cascade even in the presence of LPS, while Slpi-KO DCs fail to respond to quercetin and release high amounts of inflammatory cytokines. Our data indicate that nutritionally-derived quercetin is able to imprint Slpi expression in DCs resident in the tip of the intestinal villi, where the frequency of DC protrusions into the intestinal lumen is far greater. It is important to underline that results obtained within this study address the response of the lamina propria-resident dendritic cells to luminal quercetin, rather than the systemic effects. Additional studies are required to prove nutritional regimes enriched in quercetin can be considered important to sustain intestinal homeostasis and immunological tolerance in an Slpi-dependent manner.

## Figures and Tables

**Table 1:** Quantification of quercetin in stool samples from different regions of the intestine obtained by liquid chromatography-mass spectrometry (LC-MS). Mean values for mice fed with standard and quercetin-enriched food are expressed in µg/mL for fecal sample and ng/10 mg for ileum tissue.

Sample Analysis	Standard Food (µg/mL)	Quercetin-Enriched Food (µg/mL)
Stool	Not Detected	696.47 ± 600.13
Duodenum content	Not Detected	108.44 ± 106.18
Ileum content	Not Detected	76.44 ± 54.21
Colon content	Not Detected	49.43 ± 0.46
Ileum tissue	Not Detected	0.26 ± 0.21
